# Comparative Genomic and Metabolomic Analyses of Two *Pseudomonas aeruginosa* Strains With Different Antifungal Activities

**DOI:** 10.3389/fmicb.2020.01841

**Published:** 2020-07-31

**Authors:** Shumin Wang, Zhendong Huang, Qing Wan, Shuo Feng, Xiaochen Xie, Ruiling Zhang, Zhong Zhang

**Affiliations:** ^1^Collaborative Innovation Center for the Origin and Control of Emerging Infectious Diseases, Shandong First Medical University (Shandong Academy of Medical Sciences), Tai’an, China; ^2^School of Basic Medical Sciences, Shandong First Medical University (Shandong Academy of Medical Sciences), Tai’an, China

**Keywords:** *Pseudomonas aeruginosa*, phenazine-1, 6-dicarboxylic acid, pyocyanin, antimicrobial compounds, biological control, housefly

## Abstract

*Pseudomonas aeruginosa* isolated from the plant rhizosphere has been widely used as an effective strain in biological control against plant disease. This bacterium promotes plant growth and protect plants against various phytopathogens through the production of phenazine metabolites. In this study, the strain *P. aeruginosa* Y12 with anti-*Beauveria bassiana* activity was isolated from the gut of housefly larvae. It was comparatively analyzed with the strain *P. aeruginosa* P18, which showed no anti-*B. bassiana* activity. Genomic and metabolomic methods were used to obtain a comprehensive understanding of the antimicrobial mechanism of Y12. After whole-genome resequencing of the two strains, a total of 7,087 non-synonymous single-nucleotide polymorphisms (nsSNPs), 1079 insertions and deletions (InDels), 62 copy-number variations (CNVs) and 42 structural variations (SV) were found in both strains. We analyzed the differentially abundant metabolites between Y12 and P18, and identified six bioactive compounds that could be associated with the antimicrobial activity of Y12. Additionally, we found that, unlike other previously reported rhizospheric *P. aeruginosa* strains, Y12 could produce both phenazine-1,6-dicarboxylic acid (PDC) and pyocyanin (PYO) at significantly higher concentrations than P18. As *B. bassiana* is an effective biological insecticide that can cause high mortality in adult houseflies but has little effect on housefly larvae, we believe that *P. aeruginosa* Y12, identified in housefly larvae but not in adults, were beneficial for the development of housefly larvae and could protect them from *B. bassiana* infection through the production of toxic metabolites.

## Introduction

The Gram-negative bacterium *Pseudomonas aeruginosa* is an opportunistic pathogen that can cause severe acute and chronic infections (Bean et al., 2016; Chevalier et al., 2017). The metabolic flexibility of this bacterium makes it easier for it to adapt to complex environmental niches such as the rhizosphere, animal hosts and the hospital environment (Frimmersdorf et al., 2010; Mulcahy et al., 2014). *P. aeruginosa* has become the principal cause of death and sepsis in cystic fibrosis patients and immunocompromised individuals. It is also a major threat to patients with burn wounds, chronic wounds and chronic obstructive pulmonary disorder (Shaan and Gellatly, 2013; Berube et al., 2016).

*Pseudomonas aeruginosa* can produce phenazine metabolites, which are heterocyclic nitrogen-containing compounds, and inhibit the growth of various pathogenic microorganisms (Hariprasad et al., 2014; Prabhu et al., 2014). These phenazine derivatives from *Pseudomonas* species are a large class of secondary metabolites, such as phenazine-1-carboxylic acid (PCA), 2-hydroxyphenazine (2-OH-PHZ), phenazine-1-carboxamide (PCN), pyocyanin (l-hydroxy-5-methyl-phenazine, PYO) and pyoluteorin (Plt) (Sorensen and Klinger, 1987; Jayaseelan et al., 2014; George et al., 2015). Due to their broad-spectrum antimicrobial activity, many of these compounds have been commercially used as biocontrol agents in agriculture (Simionato et al., 2017). PCA produced by *P. aeruginosa* M18 has been developed into an effective biological fungicide, designated “Shenqinmycin,” which can promote plant growth and protect crops from pathogenic bacterial infections (Du et al., 2015; Zhou et al., 2016). PYO produced by *P. aeruginosa* strains was not only effective antifungal agent *in vitro*, but a quorum sensing controlled virulence factor acting as a policing metabolite to restrict the appearance of social cheaters *in vivo* (Castaneda-Tamez et al., 2018). In addition to rhizosphere colonization, *Pseudomonas* has also been found in the gut of insects (Akbar et al., 2018). However, there have been few reports on *P. aeruginosa* and its potential roles in insects (Indiragandhi et al., 2007). The antimicrobial activity and mechanism of this bacterium in insects need further investigation.

Entomopathogenic fungi could cause high mortality in insects by attacking and infecting their host (Indiragandhi et al., 2007; Boucias et al., 2018). Some of these fungi kill pests with little damage to crops and have been used as ecofriendly insecticides in the biological control of pests. *Beauveria bassiana* is a soilborne entomopathogenic fungus that can penetrate the cuticle and cause infections in various insects, including pine caterpillar, corn borer, thrips, whiteflies, aphids and planthopper (Butt et al., 2016; Lu et al., 2016; Ishii et al., 2017; Weeks et al., 2017; Lee et al., 2019). *B. bassiana* plays an important role in pest control (Blake and Bextine, 2002). It has been used for controlling flies, mosquitos and ticks in bait or spray form. In houseflies, application of *B. bassiana* can cause significant mortality in 7 days. However, *B. bassiana* conidium formulation can effectively kill adult houseflies but with little effect on the larvae (maggots) (Watson et al., 1995; Weeks et al., 2017).

In this study, we isolated an anti-*B. bassiana* bacterium from the gut of housefly larvae. The bacterium was identified as *P. aeruginosa*, named Y12. The strain *P. aeruginosa* Y12 was comparatively analyzed with *P. aeruginosa* P18, which was isolated from wastewater but did not show anti-*B. bassiana* activity. Whole-genome sequencing and metabolomic analyses were conducted to investigate the mechanisms underlying the different antimicrobial properties exhibited by the two *Pseudomonas* strains. Single-nucleotide polymorphisms (SNPs), insertions and deletions (InDels), structural variation (SV) and copy-number variations (CNVs) were identified across the genome. Metabolomic analysis revealed that strains Y12 and P18 could produce phenazine derivatives of phenazine-1,6-dicarboxylic acid (PDC) and PYO, which were the first reported wild-type *P. aeruginosa* strains with the ability to produce both compounds. However, the concentration of the phenazine compounds in Y12 was significantly higher than that in P18. Comparative analysis of the metabolomes of Y12 and P18 revealed higher levels of other bioactive compounds, including demeclocycline-HCl, 2-hydroxy-4-methoxyacetophenone-5-sulfate, sulfamethoxypyridazine, orientin-7-*O*-sulfate and terconazole, in Y12 than in P18, with a concentration difference of more than forty-fold. We assume that these compounds contribute to the anti-*B. bassiana* activity of *P. aeruginosa* Y12. In this study, we analyzed two different *P. aeruginosa* strains using omics approaches and suggested the beneficial role that *P. aeruginosa* Y12 would play in the resistance of housefly larvae to *B. bassiana* infection.

## Materials and Methods

### Materials

The housefly (*Musca domestica*) colony was reared and maintained in the Laboratory of Vector and Insect Diseases of Shandong First Medical University for approximately 15 years. The *P. aeruginosa* P18 strain was isolated from wastewater in the treatment plant of Taian City, Shandong Province, China (E:117.06, N36.25). *B. bassiana* was isolated from naturally infected *Cryptotympana atrata* in Bashangou in Mount Tai, Shandong Province, and named *B. bassiana* BB. *Fusarium graminearum* was the preserved strain in our lab. The phenazine compounds PYO and PCA were purchased from Xiyuan Corp. (Shanghai, China). The antibiotics levofloxacin and gentamicin sulfate were purchased from Altascientific Corp. (Tianjin, China).

### Isolation and Antifungal Activity of *P. aeruginosa*

Approximately 30 of 1- to 3-day-old larvae and adult houseflies were starved for 4 h, placed in aseptic centrifugal tubes, sterilized with 75% (v/v) ethanol for 10 min, and then rinsed 3 times with sterilized water to remove bacteria from the insect surface. After rinsing, the gut contents were extracted in a biosafety cabinet and ground manually with a glass rod. After gradient dilution, the grinding fluid was inoculated on solid beef extract peptone medium. After incubation at 37°C for 48 h, colonies on the culture medium were selected according to color, size and other characteristics, and single colonies were transferred to fresh plates for further purification until the colonies on the plate were completely consistent.

To assay the antifungal activities of the isolates, each colony was inoculated into 5 mL of LB broth in centrifuge tubes, which were shaken at 37°C for 24 h. The inhibited fungal spore germination was further assayed through a plate confrontation test. The appropriate diluted spore suspension of *B. bassiana* BB and *F. graminearum* were spotted onto potato dextrose agar medium, respectively. Four 6-mm-diameter filter papers were symmetrically placed on the agar medium, 5 μL of overnight-cultured *P. aeruginosa* Y12 or P18 was added to three filter papers, and LB medium was added to the last filter paper as a control. The plates were cultured at 28°C for 3 days. The diameters of the inhibition zone were measured to evaluate the effects of different treatments. The experiments were conducted with three independent biological replications.

### Phylogenetic Analysis

To generate a bacterial phylogenetic tree, we retrieved the universal single copy genes from each genome using OrthoFinder v2.3.11 (Emms and Kelly, 2019) with their sequencing statistics available in Supplementary Table 1. For each individual marker gene we constructed an alignment using Muscle with default parameters. We masked the alignments and filtered the low quality columns of the alignment using Zorro. Finally, we concatenated the alignments into an overall merged alignment from which we built an approximately maximum-likelihood phylogenetic tree using RAxML (v8.2.12; boostraps: 100; model: PROTGAMMALGX). The final phylogenetic tree was explored using the FigTree software (v1.4.4) (Price et al., 2010; Sen et al., 2014).

### DNA Extraction and Whole-Genome Resequencing

The genomic DNA of *P. aeruginosa* Y12 and P18 were prepared using the Genome Extraction Kit (Tiangen Biotech, Co., Beijing, China). The extracted DNA was dissolved in TE buffer (10 mM Tris–HCl and 1 mM EDTA, pH 8.0), and its concentration and purity were measured using a NanoDrop 2000 spectrophotometer (Thermo Fisher Scientific, Waltham, MA, United States). The total DNA quantity in each sample was ≥10 μg. Next, the genomic DNA was fragmented in a Covaris instrument (Woburn, MA, United States) to an average size of 250–300 nucleotides. Library preparation was done using TruSeq Nano DNA Sample Preparation Kit (Illumina, San Diego, CA, United States), and the libraries were then sequenced using an Illumina HiSeq 4000 instrument (150 bp paired end reads). Sequence-read adapters were removed using Adapter-Removal v 2.1.7 (Schubert et al., 2016). Low-quality bases were trimmed using a 5 bp sliding window, cutting once the average Phred quality scores to below 30. After trimming, if either read from a pair of reads were <50 bp in length, then both reads were discarded.

### Variant Analysis

High-quality sequence reads were mapped to the *P. aeruginosa* PAO1 reference genome (GenBank accession number: ASM676v1) using BWA v 0.7.12-r1039 (Li and Durbin, 2009). The alignments were improved using the Picard package^1^ with the following two commands: the “FixMateInformation” command was used to ensure that all mate-pair information was in sync between each read and its mate pair, and the “MarkDuplicates” command was used to mark potential PCR duplicates. Where multiple read pairs had identical external coordinates, only the pair with the best mapping quality was retained; the others were marked as PCR duplicates. We then undertook a local realignment of the mapped reads around indels using the GATK package in two steps: the “RealignerTargetCreator” command was used to determine suspicious intervals that probably need realigning, and the “IndelRealigner” command was used for realignment of such intervals. After alignment, we carried out variant calling using the Bayesian approach as implemented in the GATK package^2^. The variants were further filtered according to the following criteria: RMS mapping quality of ≥40, site quality score of ≥30, variant confidence/quality by depth of ≥5, and the reads covering a major variant were at least five times greater than that of the minor variant. Sites that failed these criteria in any strain were removed from the analysis.

### Genome Assembly, Annotation and Phylogenetic Analysis

The high-quality reads were *de novo* assembled with SPAdes genome assembler v3.13.1 (Bankevich et al., 2012). Gaps inside the scaffold were closed with GapCloser v 1.12^3^. Gene prediction was conducted by integrating *ab initio* gene predictor GeneMarkS v4.28 and homology-based gene predictor of Exonerate v2.4.7 (Besemer et al., 2001; Slater and Birney, 2005). All the independent gene sets were merged as the final gene models using EVidenceModeler v1.1.1 software (Haas et al., 2008). All newly sequenced genomes were ordered on the basis of the reference genome with Mauve v 2.3.1 (Darling et al., 2004). BLAST calculation of average nucleotide identity (ANI) values between the genomes was implemented as described by Goris et al. (2007).

### LC MS/MS Measurements

Experiments were performed in three biological replicates. Culture supernatant was harvested after 7 days cultivation. The aliquots of the culture supernatant were rapidly filtered through a 0.22 μm membrane filter, and 10 μL of the supernatant was mixed with 250 μL of chloroform:methanol:water (1:3:1, v/v) and centrifuged at 14,000 *g* for 10 min at 4°C to collect particle-free supernatant for LC-MS analysis.

For liquid chromatography, the separation was performed by Agilent 1290 UHPLC (Agilent Corp., Santa Clara, CA, United States) and Agilent ZORBAX SB-C18 (4.6 × 150 mm, 3.5 μm) column. The injection volume was 10 μl. The separation was performed at a flow rate of 0.4 mL/min under a gradient program in which mobile phase A was composed of 5 mM ammonium acetate in water containing 0.1% acetic acid (v/v) and mobile phase B was composed of 0.05% acetic acid (v/v) in acetonitrile. The gradient program was applied as follows: *t* = 0 min, 5% B; *t* = 0.25 min, 5% B; *t* = 20 min, 80% B; *t* = 23 min, 99% B; *t* = 33 min, 99.5% B; *t* = 33.1 min, 99.5% B. The stop time was 40 min. For MS, data was acquired by Agilent 6540 Q-TOF mass spectrometer (Agilent Technologies, United States) operating in the positive and negative ion mode using Agilent Jet Stream Electrospray ionization (ESI) source. The capillary voltage was set at +3800 V (positive mode) and −3800 V (negative mode) with nozzle voltages of +0 V and −0 V, respectively. Other source conditions were kept constant in all the experiments as follow: gas temperature was kept constant at 325°C, drying gas (nitrogen) was set at the rate of 10 L/min, and the pressure of nebulizer gas (nitrogen) was 35 psi. The sheath gas was kept at a flow rate of 10 L/min and was maintained at a temperature of 330°C. The voltages of the Fragmentor, Skimmer 1, and OctopoleRFPeak were 175 V, 65 V and 750 V, respectively. The scan range was adjusted to 50–1500 m/z at the acquisition rate of 2 spectra/s. MS/MS acquisition was operated in the same parameter as in MS acquisition. Collision Energy (CE) was used at 20 or 40 eV for fragmentation of the targeted compounds.

### Data Processing and Statistical Data Analysis

All mass spectral data was acquired using Agilent Mass Hunter Qualitative Analysis software (version B.05.00, Agilent Technologies, United States). To optimize feature detection and discovery, two software packages: Mass Hunter Qualitative Analysis and open-source software XCMS (version 1.38.0) operating in R, which adopted different peak detection and alignment algorithms, were used (Smith et al., 2006). For Mass Hunter Qualitative Analysis software, data preprocessing including baseline correction, noise calculations and molecular features extraction were performed with built-in small molecule extraction algorithm. Data was subsequently processed using Mass Profiler Professional (MPP) (Agilent Technologies) for peak alignment, data filtering and statistical analysis. For XCMS, raw data files were first converted to mzDATA format and peak detection were performed with centWave alogrithm in XCMS. Data was subsequently processed using XCMS for peak alignment and data filtering. MetaboAnalyst 2.0^4^ was used for statistical analysis. Further data processing including normalization, scaling and filtering were performed prior to statistical analysis in both software. Only variables that are present in at least 60% of any group and with intensity of at least 4.0E+03 were included for analysis in order to reduce noise and low abundance metabolites. The MS data were log2-transformed and mean-centered with unit variance scaling for statistical analysis. Principal component analysis and hierarchical clustering were performed for unsupervised multivariate statistical analysis. PLS-DA were performed as supervised method to identify important variables with discriminative power. PLS-DA models were validated based on multiple correlation coefficient (R2) and cross-validated R2 (Q2) in cross-validation and permutation test by applying 2000 iterations (*p* > 0.001). The significance of the biomarkers was ranked using the variable importance in projection (VIP) score (>1) from the PLS-DA model. Student’s *t*-test for comparison between P18 and Y12. *P* < 0.05 was considered to be statistically significant. Extracted ion chromatograms of potential specific metabolites identified by statistical analysis were manually viewed to confirm the differences in peak areas between P18 and Y12 samples. Metabolites were further filtered using CAMERA package in R, Mass Hunter and manual inspection to exclude possible fragments, dimers, adducts and isotopes (Kuhl et al., 2012). Specific metabolites that were detected by both MPP and MetaboAnalyst to be statistically significant were considered to be potential biomarkers.

### Metabolite Identification

MS/MS fragmentation was performed on the identified potential specific biomarkers. Identification of potential biomarkers was carried out by METLIN database^5^, Human Metabolome Database (HMDB)^6^, *E. coli* Metabolome Database (ECMDB)^7^, MassBank^8^, LipidMaps^9^ and KEGG database^10^ search using exact molecular weights or MS/MS fragmentation pattern data and literature search. For confirmation of metabolite identity using authentic chemical standard, MS/MS fragmentation pattern of chemical standard was compared with that of candidate metabolite under same LC-MS condition to reveal any matching. In case of unknown metabolites, molecular formulae were generated using Mass Profiler Professional.

### Anti-*B. bassiana* Activity Analysis of Phenazine Compounds

Compound PYO was dissolved in DMSO, and compound PCA was dissolved in sterile deionized water to prepare stock solutions (100 μg/mL). The anti-*B. bassiana* activity of PYO and PCA were evaluated using agar well diffusion test as described above. The appropriate diluted spore suspension of *B. bassiana* BB was spotted onto potato dextrose agar medium, respectively. Four 6-mm-diameter filter papers were symmetrically placed on the agar medium, 10 μL of 100 μg/mL PYO/PCA was added to three filter papers, and sterile deionized water was added to the last filter paper as a control. The plates were cultured at 28°C for 3 days. The diameter of the inhibition zones were measured to evaluate the effects of different treatments. The experiments were conducted with three independent biological replications.

### Anti-*B. bassiana* Studies With Housefly Larvae

In order to analyze the influence of Y12 and P18 to the growth of housefly larvae, housefly larvae were cultivated with different diet including moistened wheat bran mixed with different proportion of *B. bassiana*, *P. aeruginosa* and antibiotics. In the first diet (Y12 + Bb), appropriate diluted spore suspension of *B. bassiana* BB mixed with strain Y12 were added into the wheat bran in proportion of 4:6 (wt:wt). In the second diet (P18 + Bb), appropriate diluted spore suspension of *B. bassiana* BB mixed with strain P18 were added into the wheat bran in proportion of 4:6 (wt:wt). In the third diet (Ab + Bb), appropriate diluted spore suspension of *B. bassiana* BB mixed with levofloxacin (0.2%) and gentamicin sulfate (0.2%) were added into the wheat bran in proportion of 4:6 (wt:wt). In the forth diet (LB + Bb), appropriate diluted spore suspension of *B. bassiana* BB mixed with LB broth were added into the wheat bran in proportion of 4:6 (wt:wt). In each group, 10 housefly larvae were collected, cultivated in the sterile centrifuge tube covered by air-permeable gauze and fed on different artificial diet. Each group was replicated five times with a control lacking *B. bassiana* in the wheat bran. The larval mortality in different groups were recorded everyday until all of them pupated.

## Results

### Antifungal Analysis of the *P. aeruginosa* Strains Y12 and P18

**FIGURE 1:**
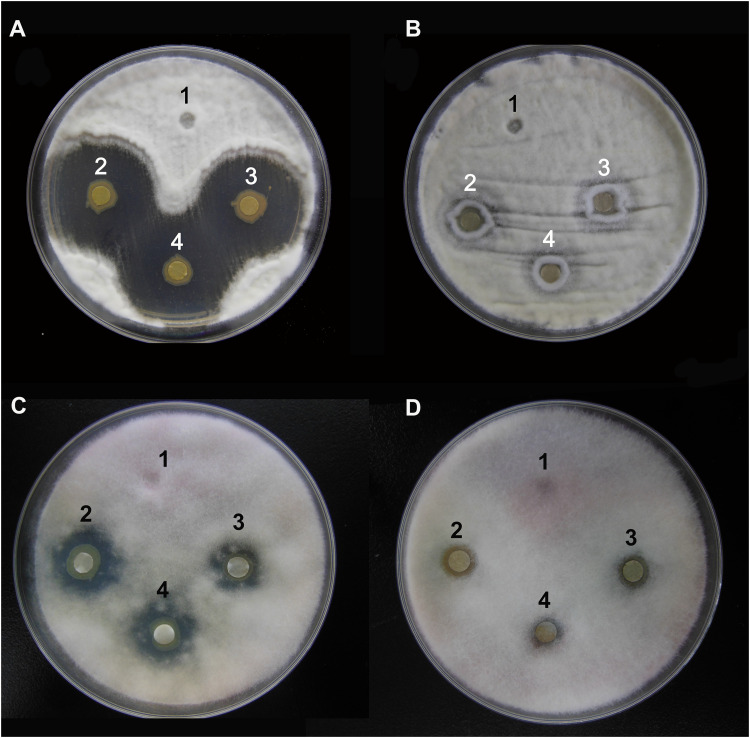
Characterization of the antifungal activity of *P. aeruginosa* strains against the growth of *B. bassiana* BB and *F. graminearum* on PDA medium. **(A)** Anti-*B. bassiana* activity of strain Y12. 1: LB medium as a control; 2–4: culture solution of strain Y12. **(B)** Anti-*B. bassiana* activity of strain P18. 1: LB medium as a control; 2–4: culture solution of strain P18. **(C)** Anti- *F. graminearum* activity of strain Y12. 1: LB medium as a control; 2–4: culture solution of strain Y12. **(D)** Anti- *F. graminearum* activity of strain P18. 1: LB medium as a control; 2–4: culture solution of strain P18.

The agar well diffusion analysis revealed that strain Y12 isolated from the intestines of housefly larvae showed strong antifungal activity against *B. bassiana* (Figure 1A). The bacteria produced obvious antifungal inhibition zones. The diameter of the inhibition zone was 28 ± 4 mm. Another strain, *P. aeruginosa* P18, did not exhibit antifungal activity (Figure 1B). Besides *B. bassiana*, strain Y12 also showed obvious antifungal activity against *F. graminearum* while P18 did not exhibit inhibitory activity (Figures 1C,D). The diameter of the inhibition zone around Y12 was 13 ± 3 mm. Compared to larvae, strains isolated from adult houseflies did not show anti-*B. bassiana* activity (data not shown).

### General Comparison of the Genomes and Phylogenetics

**FIGURE 2:**
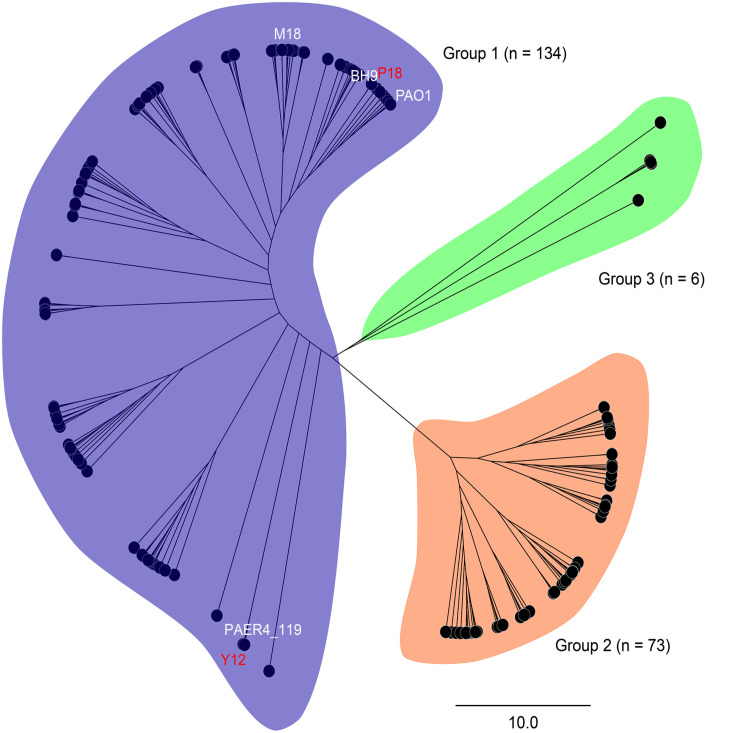
Phylogenetic tree of 213 *P. aeruginosa* strains. Strains are divided into three major groups (group 1: blue, group 2: orange and group 3: green). Sequencing statistics of the 213 *P. aeruginosa* strains were available in Supplementary Table 1.

In order to analyze the phylogenetic relationships between Y12 and P18, the genome of 213 reported *P. aeruginosa* strains were analyzed (Supplementary Table 1). Phylogenetic tree revealed three major groups among the analyzed *P. aeruginosa* strains. Group 1 (*n* = 134) and 2 (*n* = 73) included most of the analyzed strains while only 6 strains belonged to group 3 (Figure 2). Although Y12 and P18 belonged to separate sub-clusters, both of the strains were assigned to group 1. Additionally, *P. aeruginosa* PAO1 (NCBI BioProject: PRJNA331) and M18 (NCBI BioProject: PRJNA61423) which have been reported to possess broad spectrum antimicrobial activity also belonged to group 1. Phylogenetic tree showed that strain Y12 was most closely related to *P. aeruginosa* PAER4_119 (NCBI BioProject: PRJNA299511), while strain P18 clustered with the branch of *P. aeruginosa* BH9 (NCBI BioProject: PRJNA473307). However, the antimicrobial activity of strain BH9 and PAER4_119 has not been investigated till now.

### SNP, InDel, CNV and SV Detection

Genome sequencing for Y12 and P18 generated ∼9.9 and ∼9.1 million sequence reads, respectively. The genome coverage for Y12 and P18 reached 200× and 194×, respectively, compared with the *P. aeruginosa* PAO1 genome (Table 1) (NCBI bioproject: PRJNA627500). The total number of SNPs was 16,351 and 17,348 for the Y12 and P18 genomes, respectively.

**TABLE 1:** Summary of sequencing results from Y12 and P18.

**Sample name**	**Raw reads**	**Clean reads**	**Q30 (%)**	**Mapped reads**	**A_Coverage^a^**	**Ave_Fold^b^**
Y12	11,722,446	9,921,774	93.75	8,738,685	97.54%	200×
P18	10,787,230	9,174,984	97.77	8,473,940	97.73%	194×

*^*a*^A_Coverage, assembly coverage calculated as the proportion of bases in the genome assembly that were covered by at least one read. ^*b*^Ave_Fold, average fold that was calculated as the average depth of coverage across the whole genome.*

Among the genic SNPs, a total of 22,517 were synonymous SNPs, while only 7,087 were non-synonymous SNPs (nsSNPs), which could be associated with the production of antifungal metabolites. A total of 1079 InDels were identified in Y12 and P18. Among the InDels, 518 were insertions compared to the reference sequence (Table 2). In Y12, six InDels were found to be located upstream and downstream of the phenazine biosynthesis genes, including four insertions and two deletions (Table 3). One insertion, “GTTTCACGGTGGAT,” was found downstream of the *phzG2* gene (dist = 13) in Y12. Another insertion, “AACGATATC,” was found downstream of the *phzA1*-*phzB1* (dist = 5) gene and upstream of the *phzC1* (dist = 19) gene. However, no InDel was found in the intergenic and intronic regions of phenazine biosynthesis-related genes in P18.

**TABLE 2:** Summary of the detected InDels identified from Y12 and P18.

**Fields**	**Y12**	**P18**
Insertion	274	244
Deletion	313	248
UTR 5′	1	0
FrameShift	156	102
non-frameShift	69	51
UTR 3′	0	0
Total	587	492

**TABLE 3:** Information of InDels located upstream/downstream of the phenazine biosynthesis genes.

**Position**	**Gene**	**Reference**	**Alteration**
downstream	*PA1906*, *PA1907*, *phzF2*, *phzG2* (dist = 13)	–	GTTTCACGGTGGAT
upstream; downstream	*PA1906* (dist = 1); *PA1907*, *phzG2* (dist = 42)	G	–
upstream; downstream	*phzC1* (dist = 19); *phzA1*, *phzB1* (dist = 5)	–	AACGATATC
upstream; downstream	*phzC1* (dist = 14); *phzA1*, *phzB1* (dist = 10)	–	CC
upstream; downstream	*phzC1* (dist = 6); *phzA1*, *phzB1* (dist = 18)	T	–
upstream; downstream	*phzC1* (dist = 4); *phzA1*, *phzB1* (dist = 20)	–	CCC

**FIGURE 3:**
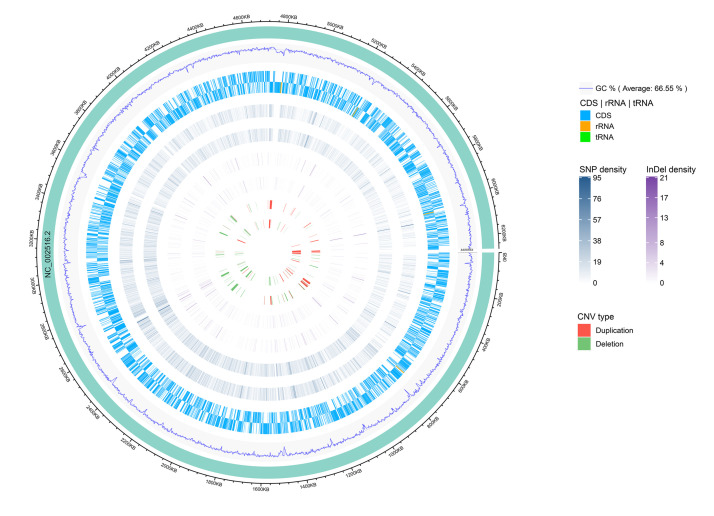
Genomic distribution of CNVs, InDels and SNPs. The innermost circle shows scatter plots of the CNVs, then from inner to outer circles shows scatter plots of the InDels, SNPs, coding region of the genome (CDS) and GC content, respectively, where the inner track represents the strain P18 and outer track represents the strain Y12. The circle outside the ring of solid colored chromosome represents the reference genome.

Among the analyzed sequences, 121 CNVs were identified, including 45 duplications and 76 deletions. The size of the CNVs ranged from 1,000 bp to 47,400 bp, and the average CNV size was 7,629 bp. The CNVs were evenly distributed along the reference genome (Figure 3). Notably, one CNV was found in the seven-gene phenazine operons (*phzA1*, *phzB1*) in Y12. In P18, no CNV was found in the genes related to phenazine compound biosynthesis.

A total of 42 SVs were found in Y12, including 7 inversions and 35 intrachromosomal transloci (ITX), and 34 SVs were found in P18, including 7 inversions and 27 ITXs. However, no SV was found in the seven-gene phenazine operons and phenazine-modifying genes. Additionally, the *qteE* gene has been reported to be an important regulatory gene in *P. aeruginosa*, and overexpression of *qteE* reduces phenazine production. We found two SNPs located upstream of the *qteE* gene in Y12 (distance: 203, C→T; distance: 194, A→G), but InDel, CNV and SV was not detected in either strain.

### Metabolite Characterization of *P. aeruginosa* Y12 Compared With the P18 Strain

**FIGURE 4:**
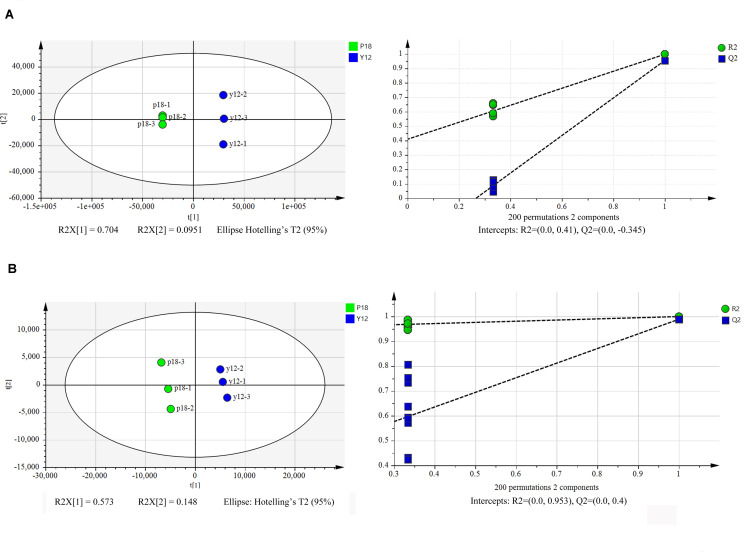
Principal component analysis score plots of the first two principal components (PC1 and PC2) for metabolites from *P. aeruginosa* Y12 and P18. **(A)** Samples analyzed by LC-MS/MS in positive ion mode from strains Y12 and P18. **(B)** Samples analyzed by LC-MS/MS in negative ion mode from strains Y12 and P18.

**FIGURE 5:**
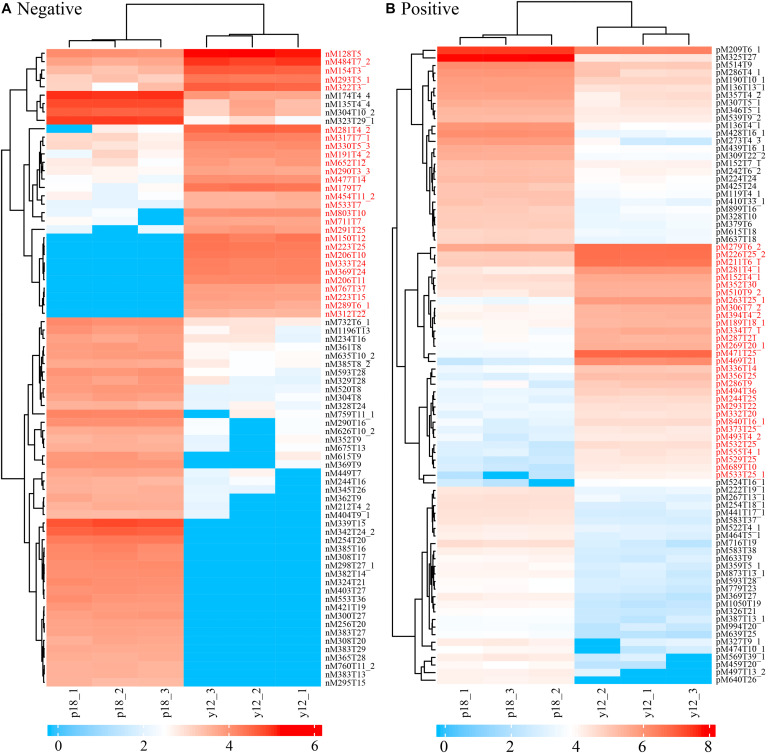
Heatmap of differentially abundant metabolites in strains Y12 and P18. **(A)** Samples analyzed by LC-MS/MS in negative ion mode from strains Y12 and P18. **(B)** Samples analyzed by LC-MS/MS in positive ion mode from strains Y12 and P18.

**FIGURE 6:**
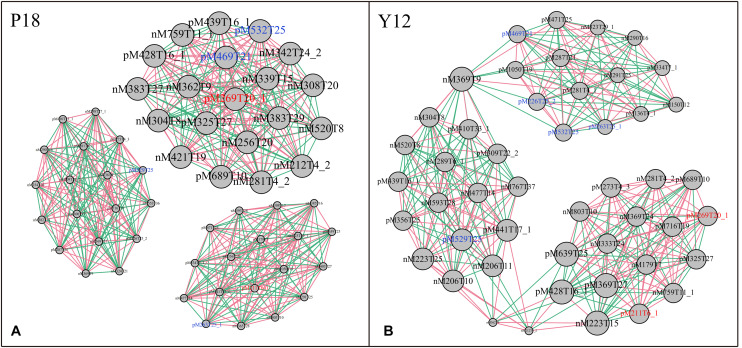
Metabolic network of differentially abundant metabolites with a more than thirtyfold difference in abundance. **(A)** Network of differentially abundant metabolites in P18. **(B)** Network of differentially abundant metabolites in Y12. The two phenazine compounds were indicated in red color: M211T6_1, pyocyanin; M269T20_1, phenazine-1,6-dicarboxylic acid; Other bioactive compounds with antimicrobial activity were indicated in blue color: M469T21, demeclocycline-HCl; M263T25_1, 2-hydroxy-4-methoxyacetophenone-5-sulfate; M226T25_2, para-(dimethylamino)-azobenzene; M529T25, orientin-7-*O*-sulfate; M532T25, terconazole.

After metabolite analysis, 22,949 compounds were found in the two strains. Among the revealed metabolites, 178 compounds were found in only Y12, 224 compounds were found in only P18, and 22,547 compounds were present in both strains. In the metabolomic results, the principal component analysis score plots showed that the metabolomes of the Y12 strain and P18 strain differed significantly in the first principal component (Figure 4). The metabolites that differed in content by more than tenfold are presented in the heatmap (Figure 5). For a detailed comparison, metabolites in the two strains with a concentration difference of more than thirtyfold (up/downregulated) were analyzed. The bioactive molecules that could be related to antifungal activity in Y12 were significantly upregulated (Table 4). Notably, PDC was 59.4-fold more abundant in Y12 than in P18. PYO was observed to be 44-fold more abundant in Y12 than in P18. Both of these metabolites were broad-spectrum antimicrobial compounds. Furthermore, demeclocycline-HCl, which has been reported to possess antimicrobial activity, was 1,912-fold more abundant in Y12 than in P18. Notably, very low amounts of demeclocycline-HCl were detected in P18. As an antifungal agent, terconazole was observed to be 42-fold more abundant in Y12 than in P18, in which it was detected at low levels. The metabolic network of differentially abundant metabolites with a more than thirty-fold difference in abundance revealed the correlations of these metabolites in Y12 and P18 (Figure 6). The differentially abundant metabolites in P18 clustered into three groups, and metabolites in each group were closely correlated. Compared to those in P18, the differentially abundant metabolites in Y12 showed correlations among different groups. The antifungal compound PDC is correlated to PYO in Y12 but not in P18.

**TABLE 4:** Putatively identified bioactive metabolites in Y12 and P18 that would be involved in the antifungal activity.

**Metabolites^a^**	**Name**	**Fold (p18 vs. y12)**	**log2fold (p18 vs. y12)**	***P*-value**	***Q*-value**	**Pdown (p18 vs. y12)**
Demeclocycline-HCl	M469T21	1912.927	–10.90157	0.00459	0.011484	Down
Ranolazine dihydrochloride	M428T16_1	394.2216	8.622863	0.001606	0.006329	Up
2-Hydroxy-4-methoxyacetophenone 5-sulfate	M263T25_1	200.783	–7.649493	6.92E−05	0.001361	Down
L-Cysteine methyl ester hydrochloride	M136T4_1	104.5138	6.707549	0.000947	0.004799	Up
N(omega)-(ADP-D-ribosyl)-L-arginine	M716T19	62.88295	5.974597	0.001948	0.007117	Up
Phenazine-1,6-dicarboxylic acid	M269T20_1	59.49369	–5.894665	9.91E−05	0.001602	Down
Para-(dimethylamino)-azobenzene	M226T25_2	57.41059	–5.843245	0.00039	0.003052	Down
Acidissiminol epoxide	M410T33_1	48.25463	5.592596	0.007898	0.01572	Up
Orotidine	M369T27	46.47523	5.53839	0.005035	0.012088	Up
Sulfamethoxypyridazine	M281T4_1	45.72306	–5.51485	0.000657	0.004012	Down
Pyocyanin	M211T6_1	44.06676	–5.461619	0.002589	0.008371	Down
Orientin-7-*O*-sulfate	M529T25	42.77544	–5.418711	0.000675	0.00407	Down
Terconazole	M532T25	42.65806	–5.414747	0.000776	0.004369	Down
Avenacin	M1050T19	38.82209	5.278806	0.000924	0.004746	Up
Antineoplastic	M290T25_1	37.18326	5.216581	0.000998	0.004924	Up
26-Glucosyl-1,3,11,22-tetrahydroxyergosta-5,24-dien-26-oate	M639T25	35.21193	5.137993	0.002499	0.008209	Up
Gummiferol	M287T21	32.92631	–5.041169	0.003131	0.00932	Down
Callichiline	M689T10	32.15789	–5.007101	0.002002	0.007232	Down

*^*a*^Putative metabolite, identified based on the exact mass and retention time.*

### Anti-*B. bassiana* Activity of Phenazine Compounds

**FIGURE 7:**
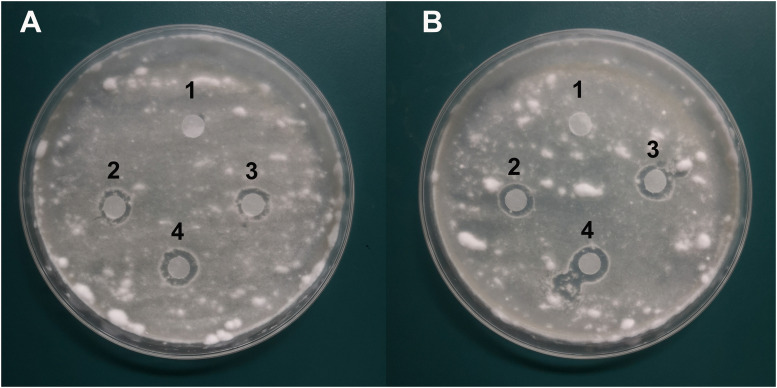
Characterization of the antifungal activity of compound PYO and PCA against the growth of *B. bassiana* BB on PDA medium. **(A)** Anti-*B. bassiana* activity of compound PYO. 1: DMSO as a control; 2–4: solution of PYO. **(B)** Anti-*B. bassiana* activity of strain PCA. 1: sterile deionized water as a control; 2–4: solution of PCA.

Antifungal analysis revealed that both PYO and PCA showed significant inhibitory activity against *B. bassiana* while the control group did not show any antifungal activity. The diameter of the inhibition zone was 9 ± 2 mm for PYO (Figure 7A) while for PCA it was 10 ± 2 mm (Figure 7B).

### Effects of Y12 and P18 on the Growth of Housefly Larvae

**FIGURE 8:**
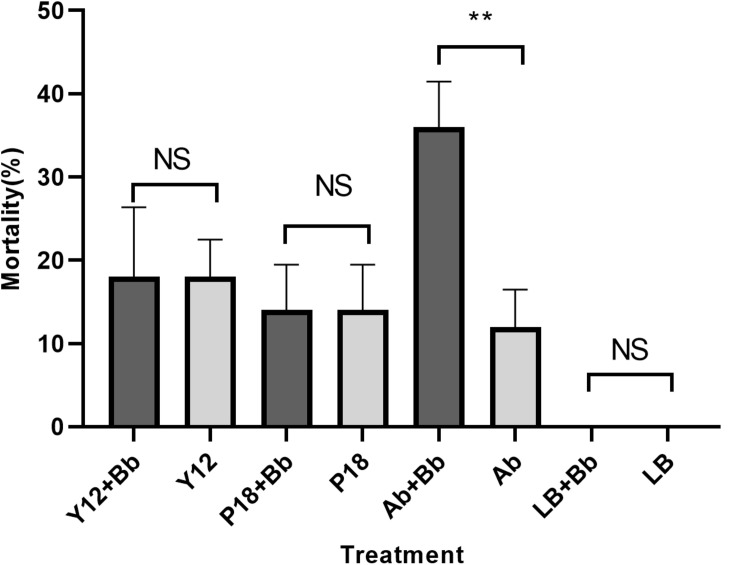
Histogram of mortality of housefly larvae after different treatment. Y12 + Bb: mortality of housefly larvae cultivated in wheat bran mixed with *B. bassiana* BB and Y12; Y12: mortality of housefly larvae cultivated in wheat bran mixed with Y12 (control group); P18 + Bb: mortality of housefly larvae cultivated in wheat bran mixed with *B. bassiana* BB and P18; P18: mortality of housefly larvae cultivated in wheat bran mixed with P18 (control group); Ab + Bb: mortality of housefly larvae cultivated in wheat bran mixed with *B. bassiana* BB and antibiotics; Ab: mortality of housefly larvae cultivated in wheat bran mixed with antibiotics (control group); LB + Bb: mortality of housefly larvae cultivated in wheat bran mixed with *B. bassiana* BB and LB broth; LB: mortality of housefly larvae cultivated in wheat bran mixed with LB (control group). Symbol denotes level of statistical significance: **, statistically significant; NS, non-significant.

Our previous study has showed that application of *B. bassiana* caused significantly higher mortality in housefly adults than in larvae (Supplementary Figure S1). As Y12 exhibited strong anti-*B. bassiana* activity, the influence of *P. aeruginosa* strains to the growth of housefly larvae during *B. bassiana* invasions was analyzed. According to the results, application of strain Y12, P18 and antibiotics all caused mortality in larvae. However, group “Ab + Bb” whose diet were added to antibiotics induced the highest mortality during *B. bassiana* infections. There was a significantly difference (*t* = 9.798, *p* = 0.0006) between group “Ab + Bb” and “Ab” (control group), indicating that addition of antibiotics increased the mortality of housefly larvae to *B. bassiana* (Figure 8).

## Discussion

In the present study, *P. aeruginosa* Y12 with antifungal activity was isolated from the gut of housefly larvae. Our results suggested that PDC and PYO are the key compounds responsible for the antifungal activity of Y12. Further analysis revealed additional bioactive metabolites that would be implicated in the antimicrobial properties of *P. aeruginosa* Y12. We believe that *P. aeruginosa* Y12 is a beneficial bacterium for housefly larvae and helps them resist entomopathogenic fungal infection.

**FIGURE 9:**
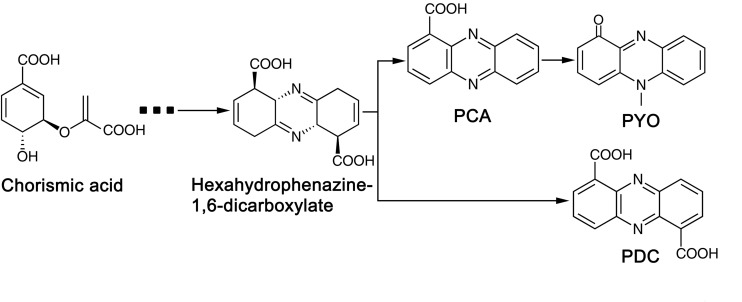
Biosynthetic pathway of phenazine compounds.

Phylogenic analysis showed that the 213 *P. aeruginosa* strains clustered to three major groups, which was consist with the previous study reported by Freschi et al. (2015). In a more recent study, phylogenetic relationships of 1311 *P. aeruginosa* isolates were comprehensively analyzed (Freschi et al., 2019). The research revealed five groups (group1, 2, 3, 4 and 5) in the phylogenetic tree and reported that the differences between five groups would be mostly due to the changes at sequence level. Among the strains they analyzed, 98% of the analyzed strains belonged to group 1–2 while 2% belonged to group 3–5. Here, in this study, 97% of the analyzed strains belonged to group 1-2, and only 6 strains belonged to group 3. As strains that belonged to group 4 and 5 were rare, we assume that strains analyzed in this study were not enough to show the 5-group phylogenetic relationship. The fungicidal activities of *P. aeruginosa* were attributed to the production of phenazine derivatives. The *phzA1B1C1D1E1F1G1* operon and *phzA2B2C2D2E2F2G2* operon have been reported to be involved in the production of PCA, while the *PhzM* and *PhzS* genes encode enzymes that function in the synthesis of PYO from the precursor PCA (Mavrodi et al., 2001). Phylogenic tree showed that, besides Y12 and P18, the antimicrobial strain PAO1 which predominantly produces PYO and strain M18 which predominantly produces PCA were also belonged to group 1. However, unlike PAO1 and M18, Y12 and P18 could produce both PDC and PYO, although the detected levels of the phenazine compounds in P18 were much lower than those in Y12. Indeed, PDC was mostly identified from *Streptomyces* species (Wang et al., 2011; Remali et al., 2017). PDC had not been widely discussed in the context of *Pseudomonas* until recently; researchers found that mutation of the *phzA* gene in *Pseudomonas chlororaphis* HT66 resulted in a significant increase in PDC production, which distinguishes the phenazine pathway producing only PCA from the pathway forming both PCA and PDC (Dasgupta et al., 2015; Guo et al., 2017; Figure 9). Another study reported the production of PDC in the genetically engineered strain *Pseudomonas chlororaphis* GP72ANlphzG (Guo et al., 2020). PDC was reported to exhibit higher antimicrobial activity than PCA (Guo et al., 2020). Therefore, strain Y12, which produces both PYO and PDC, has great potential in the field of ecofriendly pesticide application.

To analyze the gene sequences that were implicated in the synthesis of antifungal compounds. Whole-genome resequencing of Y12 and P18 was carried out using *P. aeruginosa* PAO1 as the reference genome (Holloway et al., 1994). As strain Y12 showed strong antifungal activity, the detected nsSNPs and CNV located on the phenazine synthesis operon may be key mutations that have positive impacts on the expression of enzymes involved in phenazine biosynthesis. In addition to heterocyclic phenazine metabolites, other proteins and compounds, including pyrrolnitrin, indole derivatives, chitinase, rhamnolipid, and aliphatic compounds, have also been reported to be effective antibiotics against fungal pathogens in *Pseudomonas* species (Sulochana et al., 2014; Debahuti Goswami, 2015; O’Brien and Fothergill, 2017; Liu et al., 2019). P. Indiragandhi et al. found *Pseudomonas* sp. in the gut of the diamondback moth and proposed that siderophores contribute to the antifungal activity of the strain *Pseudomonas* sp. in its host. In this study, compared to P18, strain Y12 produced significantly high amounts of phenazine compounds, which indicated their crucial role in the antifungal activity of Y12. Additionally, metabolites that exhibited significantly higher concentrations in Y12 than in P18 were also analyzed. Among the detected compounds was orientin-7-*O*-sulfate, the sulfated conjugate of orientin, which has been reported to possess antioxidant, antiaging, antiviral and antibacterial activities (Lam et al., 2016). The compound 2-hydroxy-4-methoxyacetophenone-5-sulfate is the sulfated conjugate of paeonol (2-hydroxy-4-methoxyacetophenone), which has been reported to show a wide range of bioactivities, including anti-inflammation, antioxidation and antibacterial activities (Chou, 2003). Furthermore, demeclocycline-HCl and sulfamethoxypyridazine are also antibiotics that are widely reported to be effective against various microorganisms (Mendoza et al., 1971; Chung et al., 2002). It should be noted that terconazole, the concentration of which in Y12 was 42-fold greater than that in Y18, has been reported to be an antifungal compound with broad-spectrum biocontrol activity (Weisberg, 1989). We assume that, together with phenazine compounds, the compounds reported above might also be bioactive metabolites that contribute to the biological activity of *P. aeruginosa*. Our research showed that both PYO and PCA exhibited significant *in vitro* antifungal activity which further confirmed the role of phenazine compounds played to the anti-*B. bassiana* activity of Y12. However, compound PDC which has been reported to exhibit higher antimicrobial activity has not been commercially produced till now (Guo et al., 2020). Purification and antifungal analysis of PDC would be discussed in our further research.

To date, little is known about the role of *P. aeruginosa* with antimicrobial activity in insects. A wide range of microbes inhabit the intestinal tracts of insects. These gut microbes, including pathogenic and mutualistic microorganisms, play various roles during the development of insects. In our study, *P. aeruginosa* Y12 was isolated from housefly larvae but not from adults. Additionally, *P. aeruginosa* was also detected in the samples isolated from the larval breeding environment, which indicated that some of the *P. aeruginosa* were present in the larval feces and colonized the breeding environment (Supplementary Figure S2). In larvae experiment, although addition of antibiotics and *P. aeruginosa* to the breeding environment increased the mortality of housefly larvae, it did not increase the infection rate of *B. bassiana* compared to the control group. However, it was observed that application of antibiotics which specifically targeted Gram-negative bacteria induced higher mortality in housefly larvae, which suggested that removal of *P. aeruginosa* increased the probability of *B. bassiana* infection. Therefore, we believe that *P. aeruginosa* colonizing the larval breeding environment released metabolic compounds that were virulent to *B. bassiana* and therefore protected housefly larvae from entomopathogenic fungal infections. In turn, the host larvae provided an appropriate biological environment for *P. aeruginosa* colonization. In contrast to strain Y12, strain P18 isolated from wastewater did not exhibit anti-*B. bassiana* activity. We proposed that functional coevolution had occurred between the insect-colonized strain *P. aeruginosa* Y12 and the housefly larvae, leading to the metabolic difference between Y12 and P18.

In conclusion, our results revealed that *P. aeruginosa* Y12 with antifungal activity plays an important role in housefly larvae and helps them resist *B. bassiana* invasion. Our research provide insight into the role that *P. aeruginosa* might played in insect during pathogenic fungal invasions. The strain *P. aeruginosa* Y12, which produces various antimicrobial compounds, has great potential for applications in the biological control of pathogenic fungi in both insects and plants.

## Data Availability Statement

The datasets presented in this study can be found in online repositories. The names of the repository/repositories and accession number(s) can be found at: https://www.ncbi.nlm.nih.gov/, PRJNA627500.

## Author Contributions

SW and ZH designed the study under the guidance of ZZ and RZ. SW drafted and corrected the manuscript under the guidance of ZZ. SW, ZH, QW, SF, and XX carried out the experiments. All authors approved the final manuscript.

## Conflict of Interest

The authors declare that the research was conducted in the absence of any commercial or financial relationships that could be construed as a potential conflict of interest.

## Footnotes

1 http://sourceforge.net/projects/picard/

2 https://software.broadinstitute.org/gatk/

3 https://help.rc.ufl.edu/doc/GapCloser

4 www.metaboanalyst.ca

5 http://metlin.scripps.edu/

6 http://www.hmdb.ca/

7 http://www.ecmdb.ca/

8 http://www.massbank.jp/

9 http://www.lipidmaps.org/

10 http://www.genome.jp/kegg
