# Supplementary figures and images for: Comparative Genomic and Metabolomic Analyses of Two Pseudomonas aeruginosa Strains With Different Antifungal Activities
Source: Front Microbiol. 2020 Jul 31;11:1841. doi: 10.3389/fmicb.2020.01841 (PMC7412747; doi:10.3389/fmicb.2020.01841)

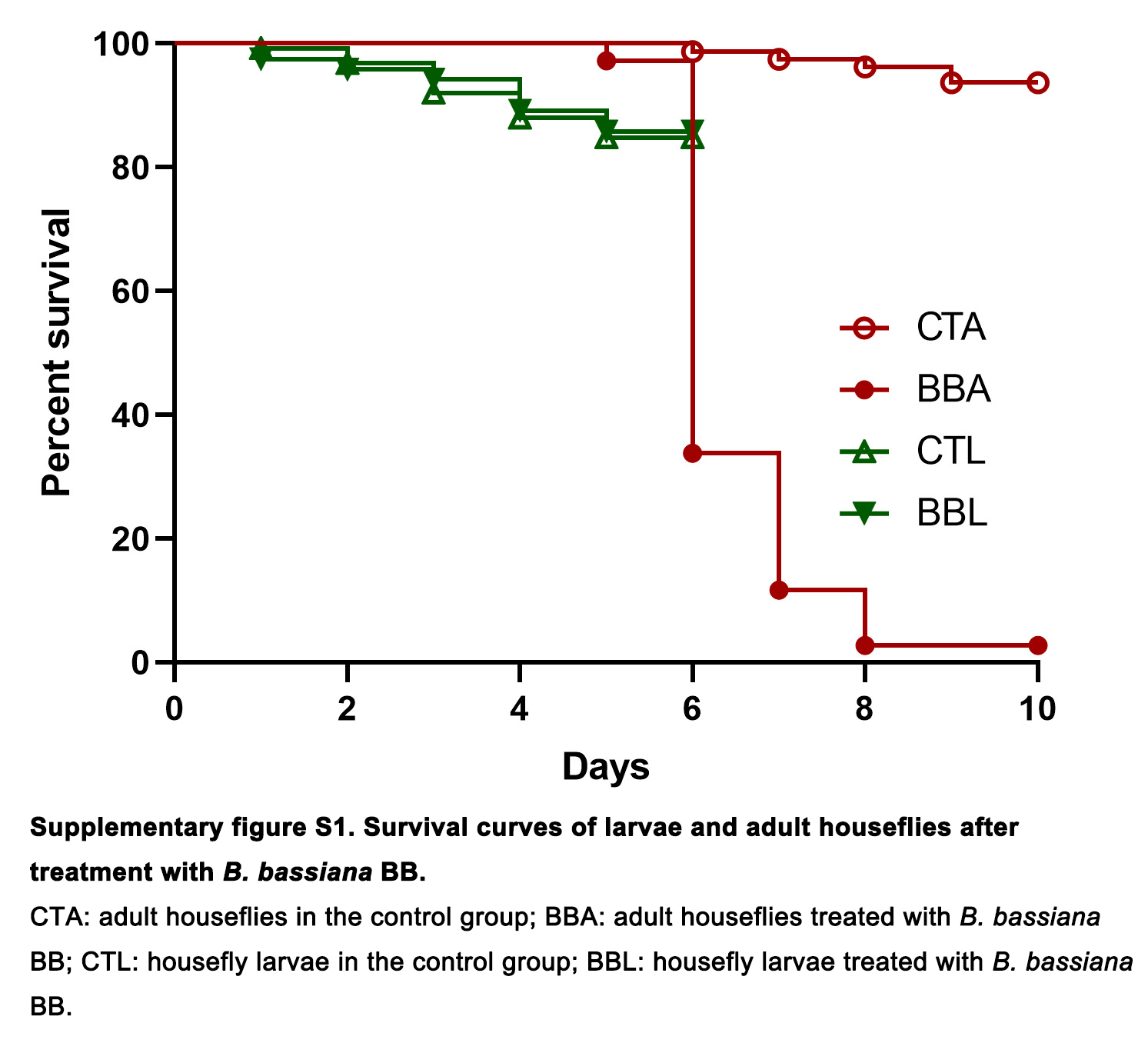

Supplement: Supplementary file 1 [file Image_1.TIF]

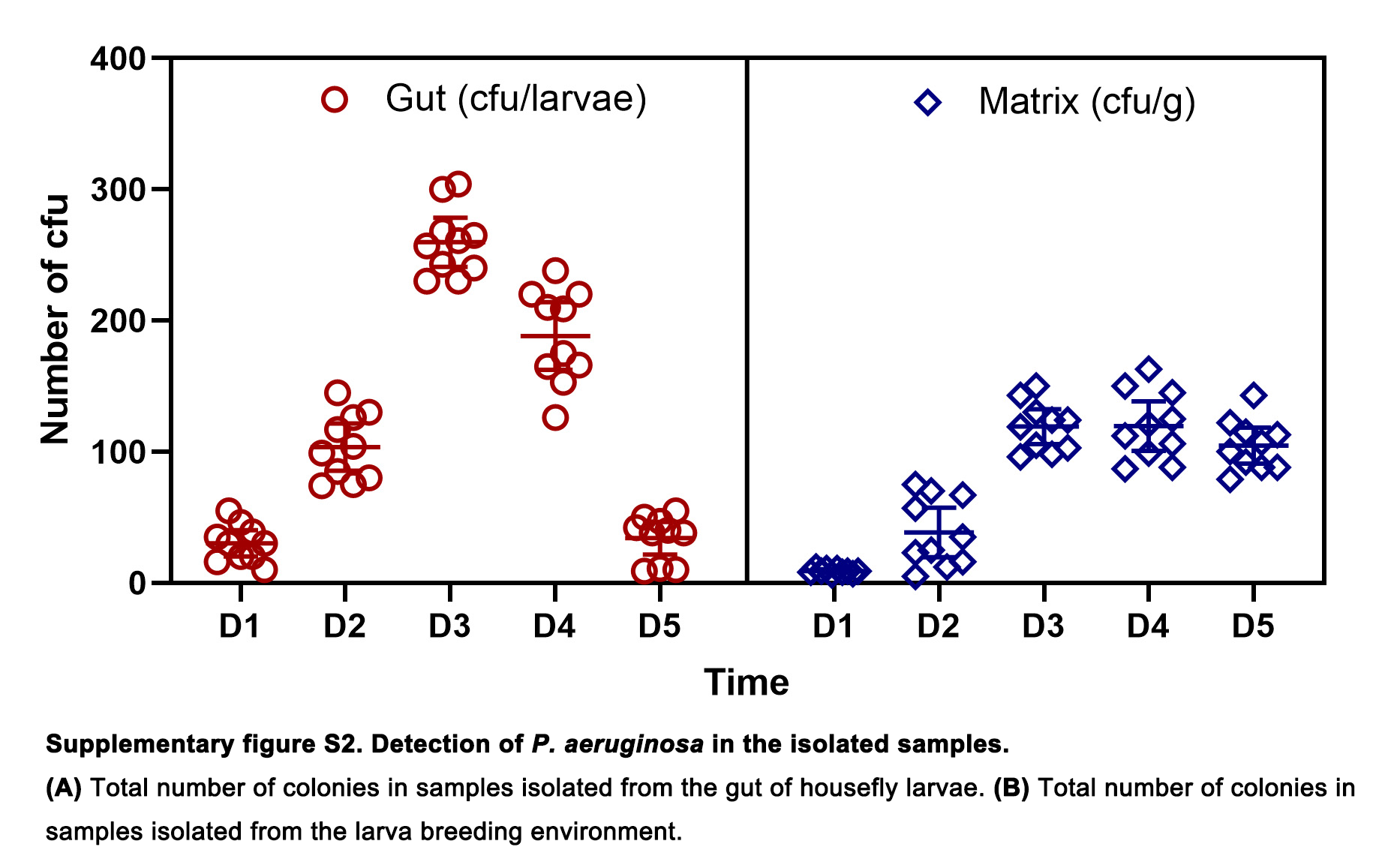

Supplement: Supplementary file 2 [file Image_2.TIF]
